# Evaluation of Phenotypic Tests to Detect Extended-Spectrum β-Lactamase (ESBL)-Producing Klebsiella oxytoca Complex Strains

**DOI:** 10.1128/jcm.01706-22

**Published:** 2023-03-13

**Authors:** Edgar I. Campos-Madueno, Aline I. Moser, Peter M. Keller, Vincent Perreten, Laurent Poirel, Patrice Nordmann, Andrea Endimiani

**Affiliations:** a Institute for Infectious Diseases, University of Bern, Bern, Switzerland; b Graduate School of Cellular and Biomedical Sciences, University of Bern, Bern, Switzerland; c Institute of Veterinary Bacteriology, University of Bern, Bern, Switzerland; d Medical and Molecular Microbiology, Department of Medicine, University of Fribourg, Fribourg, Switzerland; e National Reference Center for Emerging Antibiotic Resistance (NARA), Fribourg, Switzerland; Johns Hopkins University

**Keywords:** *Klebsiella oxytoca*, confirmatory test, detection, ESBL, OXY, CTX-M, avibactam, CDT, DDST, clavulanate

## Abstract

Klebsiella oxytoca complex (*Ko*C) species may overproduce their chromosomal class A OXY β-lactamases, conferring reduced susceptibility to piperacillin-tazobactam, expanded-spectrum cephalosporins and aztreonam. Moreover, since clavulanate maintains its ability to inhibit these enzymes, the resulting resistance phenotype may falsely resemble the production of acquired extended-spectrum β-lactamases (ESBLs). In this work, a collection of 44 *Ko*C strains of human and animal origin was characterized with whole-genome sequencing (WGS) and broth microdilution (BMD) susceptibility testing. Comparison of ESBL producers (*n* = 11; including CTX-M-15 [*n* = 6] and CTX-M-1 [*n* = 5] producers) and hyperproducers of OXYs (*n* = 21) showed certain phenotypic differences: piperacillin-tazobactam (MIC_90s_: 16 *versus* >64 μg/mL), cefotaxime (MIC_90s_: 64 *versus* 4 μg/mL), ceftazidime (MIC_90s_: 32 *versus* 4 μg/mL), cefepime (MIC_90s_: 8 *versus* 4 μg/mL) and associated resistance to non-β-lactams (e.g., trimethoprim-sulfamethoxazole: 90.9% *versus* 14.3%, respectively). However, a clear phenotype-based distinction between the two groups was difficult. Therefore, we evaluated 10 different inhibitor-based confirmatory tests to allow such categorization. All tests showed a sensitivity of 100%. However, only combination disk tests (CDTs) with cefepime/cefepime-clavulanate and ceftazidime/ceftazidime-clavulanate or the double-disk synergy test (DDST) showed high specificity (100%, 95.5%, and 100%, respectively). All confirmatory tests in BMD or using the MIC gradient strip did not perform well (specificity, ≤87.5%). Of note, ceftazidime/ceftazidime-avibactam tests also exhibited low specificity (CDT, 87.5%; MIC gradient strip, 77.8%). Our results indicate that standard antimicrobial susceptibility profiles can raise some suspicion, but only the use of cefepime/cefepime-clavulanate CDT or DDST can guarantee distinction between ESBL-producing *Ko*C strains and those hyperproducing OXY enzymes.

## INTRODUCTION

Klebsiella oxytoca is an important opportunistic human pathogen responsible for many types of infections and hospital outbreaks (1–4). Nowadays, this organism has developed resistance to a wide range of antibiotic classes, including expanded-spectrum cephalosporins (ESCs) and carbapenems (5, 6). In particular, K. oxytoca strains producing extended-spectrum β-lactamases (ESBLs; mainly of CTX-M-type) are observed worldwide with prevalence rates ranging from 2% to 6% (7–10), while carbapenemase producers seem to rapidly emerge (1, 11–14). ESBL and carbapenemase producers have also been reported in clinical samples from animals (15–17), but data regarding this setting are still very scarce.

Recent genome-based taxonomic studies indicated that K. oxytoca is actually a complex of at least six different species that can be distinguished by sequencing the chromosomal class A *bla*_OXY_ β-lactamase (*bla*) genes in: *K. michiganensis* (*bla*_OXY-1_ and *bla*_OXY-5_), K. oxytoca (*bla*_OXY-2_), *K. spallanzani* (*bla*_OXY-3_ and *bla*_OXY-9_), *K. pasteurii* (*bla*_OXY-4_), *K. grimontii* (*bla*_OXY-6_) and *K. huaxiensis* (*bla*_OXY-8_) (1).

In all species of the K. oxytoca complex (*Ko*C), the *bla*_OXY_ gene is generally expressed at low level and confers resistance only to penicillins. However, mutations in the promoter region of *bla*_OXY_ are associated with gene overexpression. As a consequence, the overproduction of OXY-type β-lactamases ensures efficient hydrolysis of aztreonam (ATM), ceftriaxone (CRO) and, to some extent cefotaxime (CTX), whereas ceftazidime (CAZ) seems marginally affected (1, 18). Moreover, since class A β-lactamase inhibitors (e.g., clavulanate) maintain their ability to inhibit the OXY (formerly K1) enzymes, the resulting resistance phenotype of *Ko*C strains may falsely resemble the production of acquired ESBLs, and particularly that related to production of CTX-M-type enzymes (10, 19–24). In this context, some authors stated that the distinction between ESBL-producing *Ko*C (ESBL-*Ko*C) and OXY-hyperproducing *Ko*C (hOXY-*Ko*C) strains is not difficult when both the results of clavulanate-based confirmatory tests and the overall profile of β-lactam susceptibility are considered (21, 22). In fact, hOXY-*Ko*C are consistently resistant to piperacillin-tazobactam (PTC) and ATM, borderline resistant to CTX and cefepime (FEP), and fully susceptible to CAZ (21, 22). However, these analyses were performed in 2002–2004 when: (i) higher susceptibility cutoffs for ESCs were used (e.g., ≤8 μg/mL for CAZ) (25), (ii) the epidemiology of ESBLs was different (i.e., the TEM- and SHV-types ESBL were still frequent compared to the emerging CTX-M-types) (19, 26, 27), and (iii) the implementation of whole-genome sequencing (WGS) for precise characterization of strains was still in its infancy. Furthermore, some variants of OXY β-lactamases conferring an ESBL-like spectrum of activity are nowadays reported, especially in patients receiving a cephalosporin-based treatment (1). For instance, variants of OXY-2 harboring amino acid substitutions/indels at Ambler positions 167–169 (OXA-2-5, OXY-2-15) have been described to hydrolyze CAZ very well, a phenomenon not observed with wild-type (WT) OXY enzymes (7, 28).

The aim of this work was to identify a phenotypic-based strategy to distinguish between ESBL-*Ko*C and hOXY-*Ko*C strains. To do so, a contemporary collection of *Ko*C strains of human and animal origin was first characterized with both antimicrobial susceptibility tests and a WGS strategy. The performance of several inhibitor-based phenotypic confirmatory tests was then evaluated against our well-defined collection of strains from different origins.

## MATERIALS AND METHODS

### Bacterial strains.

A collection of 44 non-carbapenemase-producing K. oxytoca isolates initially identified at species level using the matrix-assisted laser desorption ionization-time of flight mass spectrometry (MALDI-TOF MS; Bruker) was used for the present analysis. In particular, the collection included 29 K. oxytoca isolates of human origin: 27 detected at the Institute for Infectious Diseases (Bern, Switzerland) and 2 (R1056 and R1057) in Créteil, France (7). Overall, 21 (72.4%) human strains were isolated during the period 2020–2022. Strains from animals (*n* = 15) were detected at the Institute of Veterinary Bacteriology (Bern, Switzerland) during 2008–2020 (Table 1). Notably, even though several carbapenemase-producing *Ko*C isolates were available for testing in our collection (11–13), we intentionally excluded them from the present analysis. In fact, such strains should first undergo assays for carbapenemase production (33), making the confirmatory tests to detect ESBL production meaningless.

**TABLE 1:** Molecular characteristics of the 44 strains of human (*n* = 29) and animal (*n* = 15) origin initially identified as K. oxytoca using the MALDI-TOF MS^*a*^

Strain^*b*^	Species^*c*^	Origin/sample/yr	ST	Antimicrobial resistance genes (ARGs)	Plasmid replicons^*g*^
7606.66	*K. michiganensis*	Human/Rectal swab/2020	ST210	*bla*_CTX-M-15_, *bla*_OXY-5-9_, *aadA5, dfrA17, mph(A), qnrS1, sul1*	IncFII(pKP91), IncFII(SARC14), IncFII(S), IncFII, IncFIA(HI1)
7907.29	*K. michiganensis*	Human/Rectal swab/2020	ST398	*bla*_CTX-M-15_, *bla*_OXY-1-2_, *aadA1, aph(3′)-Ia, dfrA1*	IncFII(pCRY), IncFII, IncFIB(K)
5401.38	*K. michiganensis*	Human/NA/2014	ST50	*bla*_CTX-M-15_, *bla*_OXY-1-2_, *bla*_OXA-1_, *bla*_TEM-1_, *aac(*3*)-IIa*, *aac(6′)-Ib-cr*, *aph(3′)-Ia*, *aph(3″)-Ib*, *aph(*6*)-Id*, *dfrA14*, *qnrB1*, *sul2*, *tet(A)*	-
1312240753	*K. michiganensis*	Human/NA/2014	ST50	*bla*_CTX-M-15_, *bla*_OXY-1-2_, *bla*_OXA-1_, *bla*_TEM-1_, *aac(*3*)-IIa*, *aac(6′)-Ib-cr*, *aph(3′)-Ia*, *aph(3″)-Ib*, *aph(*6*)-Id*, *dfrA14*, *qnrB1*, *sul2*, *tet(A)*	IncFIB(pHCM2)
8212.48	K. oxytoca	Human/Rectal swab/2021	ST37	*bla*_CTX-M-15_, *bla*_OXY-2-12_	repA(pKOX), IncFII(pCoo), IncFII, IncFIB(K)(pCAV1099-114), IncFIB(AP001918), Col156*, Col(MG828)
7407.04^*d*^	K. oxytoca	Human/Rectal swab/2019	ST2	*bla*_CTX-M-15_, *bla*_OXY-2-16_, *bla*_OXA-1_, *aac(*3*)-IIa, aac(6′)-Ib-cr,dfrA14*	IncFIB(pKPHS1)
15KM0222	K. oxytoca	Horse/Pus/2015	ST401	*bla*_CTX-M-1_, *bla*_OXY-2-7_*, aac(*3*)-IId, aadA5, aph(3′)-Ia, aph(3″)-Ib, aph(*6*)-Id, catA1, dfrA17, mph(A), sul1, sul2, tet(B)*	IncQ1, IncHI1B(R27), IncHI1A, IncFIA(HI1)
13KM0084	K. oxytoca	Horse/Pus/2013	ST364	*bla*_CTX-M-1_, *bla*_OXY-2-2_, *bla*_OXA-1_*, aac(*3*)-IId, aac(6′)-Ib-cr, aadA5, aph(3′)-Ia, aph(3″)-Ib, aph(*6*)-Id, catA1, dfrA17, mph(A), sul1, sul2, tet(B)*	IncQ1, IncM1, IncHI1B(R27), IncHI1A, IncFIA(HI1)
13KM1040	K. oxytoca	Horse/Pus/2013	ST364	*bla*_CTX-M-1_, *bla*_OXY-2-2_, *aac(*3*)-IId, aadA5, aph(3′)-Ia, aph(3″)-Ib, aph(*6*)-Id, catA1, dfrA17, mph(A), sul1, sul2, tet(B)*	IncQ1, IncHI1B(R27), IncHI1A, IncFIA(HI1)
KM57/09	K. oxytoca	Horse/Pus/2009	ST401	*bla*_CTX-M-1_, *bla*_OXY-2-7_*, aac(*3*)-IId, aadA5, aph(3′)-Ia, aph(3″)-Ib, aph(*6*)-Id, catA1, dfrA17, mph(A), sul1, sul2*	IncQ1, IncHI1B(R27), IncHI1A, IncFIA(HI1)
KM24/09	K. oxytoca	Horse/Uterus/2009	ST401	*bla*_CTX-M-1_, *bla*_OXY-2-7_*, aac(*3*)-IId, aadA5, aph(3′)-Ia, aph(3″)-Ib, aph(*6*)-Id, catA1, dfrA17, mph(A), sul1, sul2, tet(B)*	IncQ1, IncHI1B(R27), IncHI1A, IncFIA(HI1)
8208.45^*e*^	*K. michiganensis*	Human/LRT secretions/2021	ST410	*bla*_OXY-1-21_, *aph(3′)-Ia*	IncFIB(K)
8011.16	*K. michiganensis*	Human/LRT secretions/2021	ST52	*bla*_OXY-1-2_, *aph(3′)-Ia*	IncFIB(K)
7806.19	*K. michiganensis*	Human/Urine/2020	ST354	*bla*_OXY-1-1_, *aph(3′)-Ia*	IncFIB(K)(pCAV1099-114)
7202.30	*K. michiganensis*	Human/Blood/2019	ST409	*bla*_OXY-1-2_, *aph(3′)-Ia*	IncFIB(K)(pCAV1099-114), FII(pBK30683)
8311.01	K. oxytoca	Human/Urine/2022	ST21	*bla*_OXY-2-1_	-
8309.06	K. oxytoca	Human/Dialysis catheter/2022	ST399	*bla*_OXY-2-32_	-
8310.32	K. oxytoca	Human/LRT secretions/2022	ST36	*bla*_OXY-2-11_	IncFII(pKP91), IncFIB(K), Col440II, Col(pHAD28)*
8310.33	K. oxytoca	Human/LRT secretions/2022	ST36	*bla*_OXY-2-11_	IncFII(pKP91), IncFIB(K), Col440II, Col(pHAD28)*
8306.21^*e*^	K. oxytoca	Human/LRT secretions/2021	ST399	*bla*_OXY-2-32_	-
8108.57^*e*^	K. oxytoca	Human/Urine/2021	ST65	*bla*_OXY-2-33_	-
8111.31	K. oxytoca	Human/Urine/2021	ST241	*bla*_OXY-2-12_	IncFII(Yp), IncFIB(K)(pCAV1099-114), ColRNAI, Col440II, Col(pHAD28)*
8005.38-1	K. oxytoca	Human/Urine/2021	ST1	*bla*_OXY-2-18_	-
8005.38-2	K. oxytoca	Human/Urine/2021	ST1	*bla*_OXY-2-18_	-
7510.48	K. oxytoca	Human/Urine/2020	ST395	*bla*_OXY-2-10_	IncFIB(pECLA)
7610.07	K. oxytoca	Human/Urine/2020	ST19	*bla*_OXY-2-1_	-
7707.06^*e*^	K. oxytoca	Human/Blood/2020	ST396	*bla*_OXY-2-34_	IncFIB(K)(pCAV1099-114)
7802.78	K. oxytoca	Human/Urine/2020	ST176	*bla*_OXY-2-4_	IncFIA(HI1), Col440I*
7907.16	K. oxytoca	Human/Urine/2020	ST397	*bla*_OXY-2-6_	IncFII(pCRY)
R1056^*f*^	K. oxytoca	Human/Urine/2002	ST141	*bla*_OXA-2-14_, *bla*_TEM-1_, *aac(6′)-Ib-cr*, *aadA2b*, *dfrA1*, *sul1*, *tet(A)*	IncFII(Yp), IncFIB(pKPHS1), ColRNAI
R1057^*f*^	K. oxytoca	Human/Urine/2002	ST141	*bla*_OXA-2-5_, *bla*_TEM-1_, *aac(6′)-Ib-cr*, *aadA2b*, *dfrA1*, *sul1*, *tet(A)*	IncFII(Yp), IncFIB(pKPHS1), ColRNAI
08KM1888^*d*^	K. oxytoca	Dog/Pus/2008	ST34	*bla*_OXY-2-16,_ *aac(6′)-IIa, aadA5, aph(3″)-Ib, aph(*6*)-Id, catA1, dfrA17, tet(D)*	IncR, IncFIB(pHCM2)
8310.44^*e*^	*K. michiganensis*	Human/Vaginal swab/2022	ST35	*bla*_OXY-1-20_, *aph(3′)-Ia*	IncFIB(K)(pCAV1099-114)
7507.77	*K. michiganensis*	Human/LRT secretions/2019	ST43	*bla*_OXY-1-1_, *aph(3′)-Ia*	IncFIB(K)
ZH142-C	*K. michiganensis*	Human/Rectal swab/2019	ST183	*bla*_OXY-5-1_, *aadA1, sul1*	IncFII(Yp), IncFII(K), IncFIB(K)
17KM0578^*e*^	*K. michiganensis*	Cow/Nasal swab/2017	ST403	*bla*_OXY-1-22_, *bla*_TEM-1_, *aac(*3*)-IId, aadA2, aph(3′)-Ia, catA1, dfrA12, mph(A), sul1, tet(B)*	IncHI1B(R27), IncHI1A, IncFIB(K)(pCAV1099-114), IncFIA(HI1), Col440II, Col440I*
15090013	*K. michiganensis*	Snake/NA/2015	ST43	*bla*_OXY-1-1_	-
15A0136	*K. michiganensis*	Cow/Placenta/2015	ST405	*bla*_OXY-1-8_, *aph(3′)-Ia, aph(3″)-Ib, aph(*6*)-Id, sul2, tet(A)*	-
20M0142	*K. grimontii*	Cow/Milk/2020	ST400	*bla*_OXY-6-4_	IncFII(pKP91), IncFIA(HI1), Col440I, Col(pHAD28)
08KM1900	*K. grimontii*	Cow/Uterus/2008	ST404	*bla*_OXY-6-4_	IncHI1B(pNDM-MAR), IncFII(Yp)
15Km1352	*K. pasteurii*	Dog/NA/2015	ST402	*bla*_OXY-4-1_	IncFII(Yp)
17KM1096	K. oxytoca	Dog/Vaginal swab/2017	ST1	*bla*_OXY-2-18_	-
14/F0005	K. oxytoca	Monkey/Lung/2014	ST413	*bla*_OXY-2-2_	IncFII(Yp), IncFIB(K)(pCAV1099-114)
09KM0284	K. oxytoca	Horse/Pus/2009	ST199	*bla*_OXY-2-4_, *bla*_TEM-1_, *aac(*3*)-IId, aadA2, aph(3′)-Ia, aph(3″)-Ib, aph(*6*)-Id, catA1, dfrA12, mph(A),sul1, sul2, tet(B)*	IncQ1, IncHI1B(R27), IncHI1A, IncFIB(pKPHS1), IncFIA(HI1)

a ST, sequence type; ARGs, antimicrobial resistance genes; LRT, lower respiratory tract; -, not detected; NA, not available.

b Considering the phenotypic results (Table 2), strains have been grouped in ESBL producers (ESBL-*Ko*C; *n* = 11), hyperproducers of OXY enzymes (hOXY-*Ko*C; *n* = 21), and wildtype strains (WT-*Ko*C; *n* = 12).

c Identification at species level obtained implementing the WGS output.

d Strains 7407.04 and 08KM1888 possessed mutations encoding the Ser83Ile substitution in GyrA.

e In this strain, a new *bla*_OXY_ was detected (Institute Pasteur assigned the new numbering).

f These two strains were isolated from the same patient (R1057 after prolonged treatment with ceftazidime) (7).

g *, indicates that more than one replicon sequence type was detected.

### Whole-genome sequencing (WGS) and bioinformatics.

Genomic DNA isolations from the 44 strains were prepared with the Invitrogen PureLink Microbiome DNA purification kit (ThermoFisher Scientific) and sequenced using the NovaSeq 6000 Illumina platform as previously described (12, 43). In short, Illumina raw reads were first quality checked with FastQC (v0.11.9) and then trimmed with Trimmomatic (v0.36) to remove adaptors. Draft assemblies were generated with Unicycler (v0.4.8) following the Illumina-only assembly pipeline and quality checked with QUAST (v5.2.0).

*In silico* screening for antimicrobial resistance genes (ARGs) and replicon sequences was done with the ResFinder v4.1 and PlasmidFinder v2.1 (50% minimum percentage identity) software of the Center for Genomic Epidemiology (CGE; https://www.genomicepidemiology.org/), respectively. Multilocus sequence typing (MLST) was done with MLST v2.0 (CGE) and with the K. oxytoca species complex typing database (PubMLST; https://pubmlst.org/organisms/klebsiella-oxytoca). Accurate species confirmation was conducted with the Type Strain Genome Server (https://tygs.dsmz.de/). The *bla*_OXY_ genes were annotated according to the Klebsiella locus/sequence definitions database from the Institut Pasteur (BIGSdb-Pasteur; https://bigsdb.pasteur.fr/). To characterize the promoter sequences, the draft assemblies were annotated with Prokka (v1.13) and the contigs containing the *bla*_OXY_ were extracted with a custom R v4.1.2 script (seqinr package v4.2-16). The upstream regions (-33 to -32 bp) of the *bla*_OXY_ was manually scanned for the -35 (TTGTCA), 17 bp spacer and -10 (GATAGT, GATAAT, TATAGT, and TATACT) promoter sequences (18, 44). Unless specified, all bioinformatics steps above were done with default parameters.

### Antimicrobial susceptibility tests (ASTs).

Strains confirmed as *Ko*C by using the WGS output underwent ASTs implementing the ESB1F and GNX2F broth microdilution Sensititre panels with Mueller-Hinton (MH) broth (Thermo Scientific) according to the manufacturer's instructions. ASTs were performed in duplicate leading to consistent results (therefore only one MIC value was shown in Table 2). ATCC strains Escherichia coli 25922 and Klebsiella quasipneumoniae ATCC 700603 were used as controls. MICs for antibiotics were interpreted according to the 2022 European Committee on Antimicrobial Susceptibility Testing (EUCAST) criteria (40). For minocycline and cefoxitin, the Clinical and Laboratory Standards Institute (CLSI) criteria of 2022 were used (39). We defined the strains as hOXY-*Ko*C those with an MIC of CRO ≥2 μg/mL and lacking genes encoding ESBLs (*bla*_ESBLs_) or plasmid-mediated AmpCs (*bla*_pAmpCs_).

**TABLE 2:**
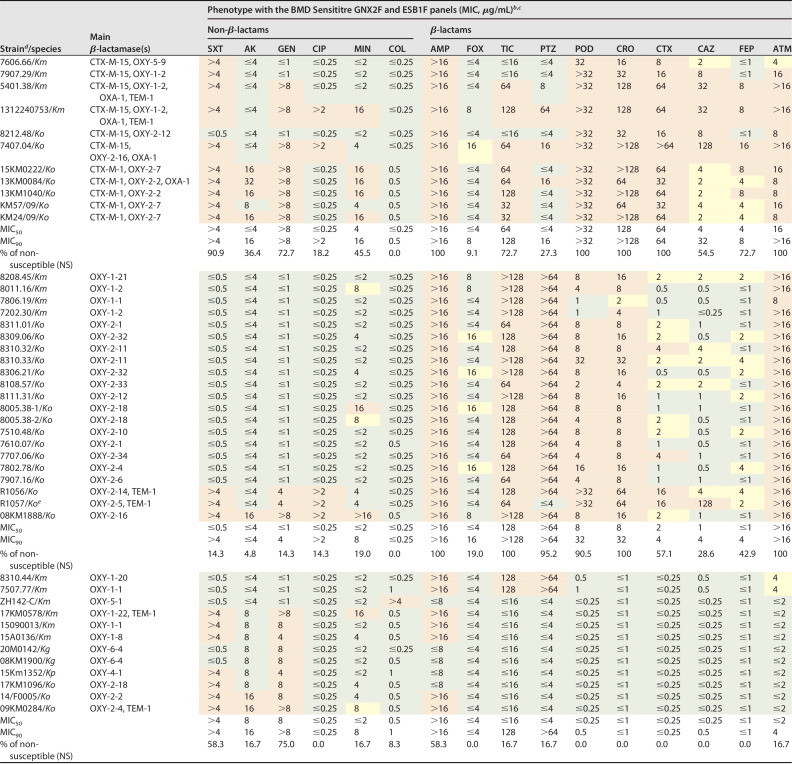
Antimicrobial susceptibility tests (ASTs) in broth microdilution (BMD) for the 44 K. oxytoca complex (*Ko*C) strains (according to the whole-genome sequencing data). Results are grouped in ESBL producers (ESBL-*Ko*C; *n* = 11), hyperproducers of OXY enzymes (hOXY-*Ko*C; *n* = 21), and wild-type strains (WT-*Ko*C; *n* = 12)^*a*^

a *Km*, *K. michiganensis*; *Ko*, K. oxytoca; BMD, broth microdilution; SXT, trimethoprim-sulfamethoxazole; AK, amikacin; GEN, gentamicin; CIP, ciprofloxacin; MIN, minocycline; COL, colistin; AMP, ampicillin; FOX, cefoxitin; TIC, ticarcillin-clavulanate; PTZ, piperacillin-tazobactam; POD, cefpodoxime; CRO, ceftriaxone; CTX, cefotaxime, CAZ, ceftazidime; FEP, cefepime; ATM, aztreonam.

b Results interpreted according to the EUCAST 2022 criteria (https://www.eucast.org/clinical_breakpoints): susceptible (green), susceptible, increased exposure (yellow), and resistant (red). Notably, all strains were fully susceptible to carbapenems (i.e., MICs for imipenem, meropenem, and ertapenem ≤0.5 μg/mL, ≤1 μg/mL, ≤0.25 μg/mL, respectively).

c EUCAST criteria for minocycline and cefoxitin are not available. Therefore, the CLSI 2022 criteria were implemented (39): susceptible (green), susceptible dose dependent or intermediate (yellow), and resistant (red).

d Strains without *bla*_ESBLs_ and *bla*_pAmpCs_ were defined as hOXY-*Ko*C or WT-*Ko*C if the CRO MIC was ≥2 or ≤1 μg/mL, respectively.

e Compared to the sequence of the OXY-2, OXA-2-5 shows a Pro167Ser substitution that confers a stronger ability to hydrolyze CAZ (7).

### Phenotypic confirmatory tests for ESBL production.

Based on the AST results, all *Ko*C strains nonsusceptible (NS) to CRO (MIC ≥2 μg/mL) were further analyzed with several inhibitory-based confirmatory tests to detect ESBL producers. As for the ASTs, these assays were repeated two times leading again to consistent results (therefore, only one value was shown in Table 3).

**TABLE 3:**
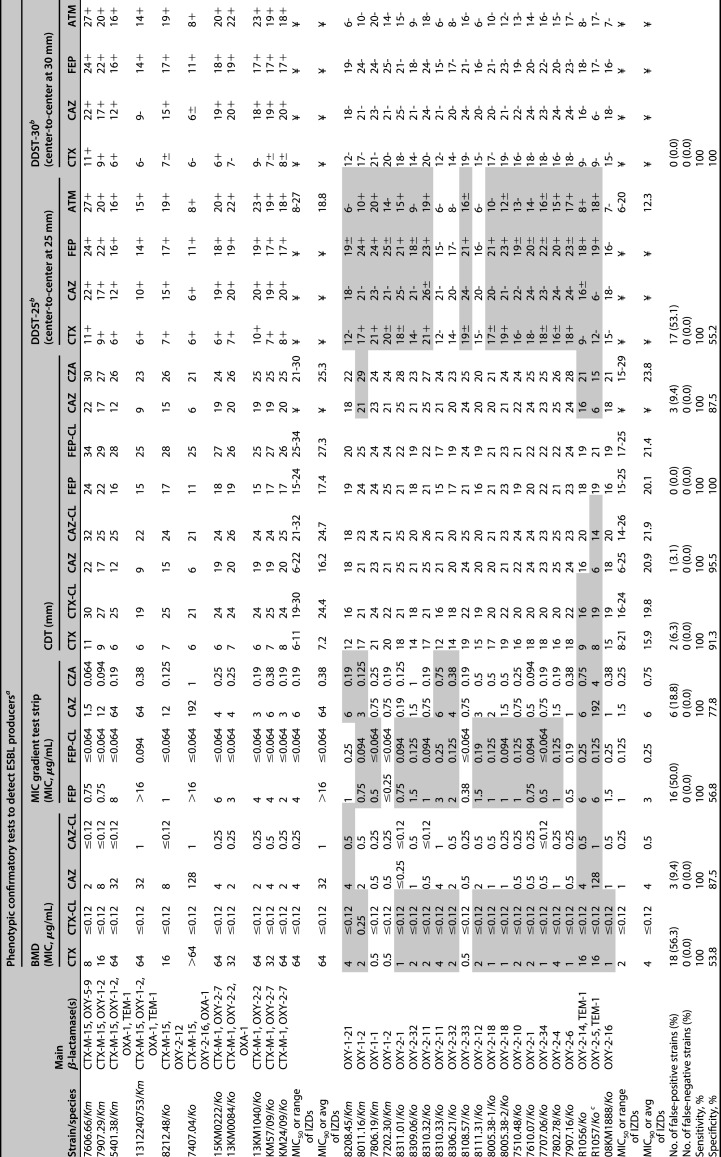
Phenotypic confirmatory tests for the K. oxytoca complex (*Ko*C) strains: comparison of the ESBL producers (ESBL-*Ko*C; *n* = 11) *versus* those hyperproducing OXY enzymes (hOXY-*Ko*C; *n* = 21) and overall performance

a False-positives are highlighted in gray. Notably, no false-negative ESBL producers were recorded with all assays. *Km*, *K. michiganensis*; *Ko*, K. oxytoca; BMD, broth microdilution; CDT, combination-disk test; DDST, double-disk synergy test; CTX, cefotaxime; CTX-CL, cefotaxime-clavulanate; CAZ, ceftazidime; CZA, ceftazidime-clavulanate; FEP, cefepime; FEP-CL, cefepime-clavulanate; IZD, inhibition zone diameter; ¥, data reported in previous sections of the present table.

b Synergy results are shown with “+” (synergy with clavulanate), “-,” no synergy; “±” weak or difficult to interpret synergy results (see example in Fig. S1). Overall, a positive result for the DDST was defined when at least 1 of the 4 β-lactam antibiotics showed synergy (“+” or “±”) with clavulanate.

c Compared to the sequence of the OXY-2, OXA-2-5 shows a Pro167Ser substitution that confers a stronger ability to hydrolyze CAZ (7).

The performance of two broth microdilution (BMD) tests was extrapolated from the results of the MIC ESB1F Sensititre panel: CTX/CTX-clavulanate (CTX-CL) and CAZ/CAZ-clavulanate (CAZ-CL). Moreover, MIC gradient strip tests (Liofilchem) with FEP/FEP-clavulanate (FEP-CL), CAZ alone and CAZ-avibactam (CZA) alone were assessed on MH agar plates (Oxoid). The results of these 4 MIC confirmatory assays were interpreted as ESBL-positive if the strain in the presence of the inhibitor had a ≥8-fold (or ≥3 2-fold) MIC decrease compared with the MIC of the cephalosporin alone (33, 39).

Four combination-disk tests (CDTs) on MH agar plates (Oxoid) were also assessed. In particular, we used the EUCAST ESBL Disk kit (Liofilchem) that includes six disks: CTX (5 μg)/CTX-CL (5/10 μg), CAZ (10 μg)/CAZ-CL (10/10 μg), and FEP (30 μg)/FEP-CL (30/10 μg). Moreover, a CZA disk (10/4 μg; Liofilchem) was also tested. Results of each of the four CDTs were interpreted as ESBL-positive if a ≥5 mm increase in the inhibition zone diameter was recorded for the cephalosporin plus inhibitor compared to the cephalosporin alone (33, 39).

Finally, *Ko*C strains were studied with the double-disk synergy test (DDST) on MH agar plates with disks of CTX (5 μg; Liofilchem), CAZ (10 μg; Liofilchem), FEP (30 μg; Liofilchem) and ATM (30 μg; Bio-Rad) placed with a distance center-to-center of 25 mm (DDST-25) and 30 mm (DDST-30) around a disk of amoxicillin-clavulanate (AMC; 20/10 μg; Bio-Rad). An ESBL-positive result was indicated when the inhibition zone around at least one of the cephalosporins or ATM disks expanded or there was a keyhole toward the AMC disk (33).

### Data availability.

The draft genome assemblies are deposited in GenBank under BioProject PRJNA894995.

## RESULTS AND DISCUSSION

The clavulanate-based phenotypic confirmatory tests show good performance and reliable results in detecting ESBL-producing E. coli and K. pneumoniae strains. In contrast, such assays resulted in high false-positive rates when performed with hOXY-*Ko*C strains (10, 19–24). It should also be noted that level of identity at the amino acid and at the nucleotide levels of those OXYs may generate false-positive results with immunochromatographic or PCR-based assays designed to detect CTX-M ESBLs, respectively (29, 30). In addition, although being faster, these non-phenotypic tests are more expensive, making their implementation limited to the screening of suspected carbapenemase producers (31, 32).

The scope of our study was to use a well-defined collection of *Ko*C strains to find a possible phenotypic-based strategy to ensure the identification of ESBL producers among those that are ESC-NS. From an epidemiological point of view, the correct detection of such strains can help to accurately define their prevalence. Furthermore, since *bla*_ESBLs_ are transferable on mobile genetic elements, separation of ESBL-*KoC* and hOXY-*KoC* strains has important public health and infection control implications (e.g., isolation measures and consequent costs) (33). This is particularly true in countries with a relatively low prevalence of carbapenemase producers (e.g., Switzerland) that still implement such rules for ESBL producers, especially those belonging to Klebsiella spp. (32).

### Molecular features of *Ko*C strains.

Based on the WGS analysis, the 44 isolates were mainly identified as K. oxytoca (*n* = 27; 61.4%) and *K. michiganensis* (*n* = 14; 31.8%) species (Table 1).

In total, 11 ESBL-*Ko*C strains were identified: 6 of human origin harbored *bla*_CTX-M-15_, while 5 from animals possessed *bla*_CTX-M-1_. Most ESBL producers possessed various plasmid-mediated ARGs against different classes of antibiotics and 4 of them also co-carried the *bla*_OXA-1_ that encodes a β-lactamase conferring resistance to PTC (Table 1) (34). Analogous data regarding the molecular characteristics of ESBL-*Ko*C strains are scarce. Of note, most of the reported human isolates possessed the *bla*_CTX-M-15_ or *bla*_SHV-12_ ESBL encoding genes (2, 35, 36), while those of animal origin carried *bla*_DHA-1_, *bla*_CTX-M-9_, *bla*_CTX-M-15_, or *bla*_SHV-12_ (15, 16). However, in these studies, characterization of *bla*_ESBLs/pAmpCs_ was obtained using only PCR-based methods and identification was generically reported as K. oxytoca. Moreover, only two surveys reported the corresponding sequence types (STs) of the ESBL-*Ko*C strains as we have done in the current study (33, 35). This lack of high-quality typing was also evident in studies evaluating the performance of phenotypic confirmatory tests for ESBL detection (see below) (10, 19–24, 37).

The remaining 33 *Ko*C strains in our collection did not possess any *bla*_ESBL_ or *bla*_pAmpC_ gene. Based on the MIC of CRO, 21 of these strains were categorized as hOXY-*Ko*C, while the last 12 isolates were defined as WT *Ko*C (WT-*Ko*C) strains for simplicity. Overall, both hOXY-*Ko*C and WT-*Ko*C strains possessed much less ARGs compared to ESBL producers. Notably, most hOXY-*Ko*C were isolated from clinical samples of humans who were hospitalized, whereas WT-*Ko*C strains were mainly detected in animals admitted from the community (Table 1).

Numerous OXY-types were detected in the overall collection of 44 *Ko*C strains, including five newly reported (Table 1). Of note, strain R1057 hyperproduced OXY-2-5, a previously described variant of OXY-2 (Pro167Ser) that hydrolyzes CAZ at much higher level than the WT OXYs (7).

The promoter region of all *bla*_OXY_ genes detected in the 44 strains was also characterized (Table S1). All of the ESBL-*Ko*C strains (*n* = 11) possessed the WT promoter (-10: GATAGT), whereas hOXY-*Ko*C strains carried three strong (-10) promoter combinations: TATAGT (*n* = 3), TATACT (*n* = 2) and GATAAT (*n* = 16). The WT-*Ko*C strains also possessed the WT promoter, except for two strains of human origin (8310.44 and 7507.77) that carried a strong promoter (-10: GATAAT) (18). Such strains were, in fact, non-susceptible to ticarcillin-clavulanate, PTC and ATM, though their respective MICs of CRO were ≤1 μg/mL without a clear explanation (Table 2).

Overall, we emphasize that previous studies analyzing the susceptibility of *Ko*C strains and the performance of phenotypic confirmatory tests for ESBL production did not provide an accurate molecular characterization as we did in the present work (10, 19–24, 37). Such information is essential to interpret the overall phenotypic and confirmatory test results illustrated below.

### Phenotypic characteristics of *Ko*C strains.

Looking at the results of the ASTs (Table 2), we first noted that, consistently with the genotypic data, ESBL-*Ko*C strains showed a frequency of associated resistance to non-β-lactam antibiotics higher than the hOXY-*Ko*C isolates. This was particularly true for trimethoprim-sulfamethoxazole (SXT) and gentamicin (GEN): 90.9% *versus* 14.3% and 72.7% *versus* 14.3%, respectively. However, this phenomenon was not sufficient to clearly discriminate between the two groups of ESC-non-susceptible *Ko*C (ESC-NS-*Ko*C) strains.

Compared, ESBL-*Ko*C and hOXY-*Ko*C strains also showed some differences in term of susceptibility profiles: PTC (MIC_90s_: 16 *versus* >64 μg/mL; NS: 27.3% *versus* 95.2%), CTX (MIC_90s_: 64 *versus* 4 μg/mL; NS: 100% *versus* 57.1%), CAZ (MIC_90s_: 32 *versus* 4 μg/mL; NS: 54.5% *versus* 28.6%), and FEP (MIC_90s_: 8 *versus* 4 μg/mL; NS: 72.7% *versus* 42.9%, respectively). Nevertheless, even this information was not useful for establishing a strategy to distinguish the two groups of ESC-NS-*Ko*C isolates. We further emphasize that the strain producing the variant OXY-2-5 (R1057) displayed a phenotype almost identical to 8 out of 11 CTX-M-producing *Ko*C strains (i.e., susceptible to PTC, non-susceptible to CTX and CAZ, and co-resistant to SXT) (Table 2).

Overall, our data indicate that phenotypic results for PTC, ESCs and ATM cannot be used to distinguish between contemporary ESBL-*Ko*C and hOXY-*Ko*C strains. Special attention should be made to PTC and CAZ (Table 2). Three ESBL-*Ko*C strains were in the resistant range for PTC because they coproduce the OXA-1 β-lactamase, whereas R1057 was fully susceptible (MIC ≤4 μg/mL) to the drug. Moreover, 4 ESBL-*Ko*C of animal origin were only moderately resistant to CAZ (MICs of 2–4 μg/mL) because they produce the CTX-M-1 that does not significantly hydrolyze this substrate (38).

### Performance of phenotypic confirmatory tests.

Since a clear distinction between ESBL-*Ko*C and hOXY-*Ko*C strains based on the ASTs was difficult, we further evaluated the performance of 10 different inhibitor-based confirmatory tests for ESBL detection.

As shown in Table 3, none of the confirmatory tests resulted in false-negative results with the 11 ESBL-*Ko*C strains (sensitivity, 100%). In particular, all assays provided results without any ambiguity (i.e., higher than the cutoffs used to define a strain as ESBL-positive) (33, 39). Consistently, this high sensitivity was noted by numerous authors implementing various confirmatory assays and also testing strains producing non-CTX-M-type ESBLs (e.g., SHV-12 and TEM-types) (20–24, 37). On the other hand, our study showed that CTX/CTX-CL BMD test, FEP/FEP-CL gradient strip test and DDST-25 gave a very high number of false-positive results when tested against hOXY-*Ko*C strains (specificity of 53.8%, 56.8%, and 55.2%, respectively). Both the CAZ/CAZ-CL BMD test and the CTX/CTX-CL CDT performed better, but still showed less than ideal specificity (87.5% and 91.3%, respectively).

The low specificity that we recorded for the FEP/FEP-CL gradient strip test when implemented for ESC-NS-*Ko*C strains was already reported by others (19–22). The same authors also observed an overall low performance for other gradient strips (i.e., CAZ/CAZ-CL and CTX/CTX-CL) that were not evaluated in the present study (19–22). Regarding the BMD-based confirmatory tests, two different studies used the MicroScan ESBL confirmatory panel to evaluate a total of 7 ESBL- (of which 6 producing CTX-Ms) and 9 hOXY-*Ko*C strains. As a result, the CAZ/CAZ-CL assay showed 100% sensitivity and specificity, whereas for the CTX/CTX-CL they were 100% and 69.2%, respectively (24, 37).

Our analysis indicated that the best performance in detecting ESBL-*Ko*C strains was achieved with the FEP/FEP-CL CDT and the DDST-30 (100% specificity for both), but also the CAZ/CAZ-CL CDT showed an acceptable specificity of 95.5% (Table 3; Fig. S1).

Previous data regarding the performance of specific CDTs and DDSTs in the context of *Ko*C is lacking. Sturn et al. tested 4 ESBL- (all TEM-types) and 17 hOXY-*Ko*C strains with the CAZ/CAZ-CL and CTX/CTX-CL CDTs resulting in a sensitivity and specificity of 100% and 85%, respectively. However, despite the good performance, the results were a combination of the two CDTs. Interestingly, the 3 false-positives hOXY-*Ko*C observed in that latter study produced the OXY-2-5 variant (Pro167Ser in OXY-2) (19). In another study, Wiegand et al. used a collection of 5 ESBL- (including 4 producing CTX-Ms) and 9 hOXY-*Ko*C strains to evaluate the performance of four CDTs: CAZ/CAZ-CL, CTX/CTX-CL, cefpodoxime/cefpodoxime-clavulanate, and cefpirome/cefpirome-clavulanate. Combining all CDTs, the authors reported 80% sensitivity and 88.9% specificity. In the same study, a DDST (CAZ, CTX, cefpodoxime and cefpirome disks placed at 25 to 30 mm away from AMC) showed overall sensitivity and specificity of 80% and 55.6%, respectively (20). However, in those two above-mentioned analyses, CTX (30 μg) and CAZ (30 μg) CLSI-recommended disks were implemented (39), whereas in the present work disks of CTX (5 μg) and CAZ (10 μg) have been used, as suggested by the EUCAST (33, 40). Therefore, a comparison with our results does not seem meaningful.

Finally, for the very first time, we assessed the performance of gradient strips with CAZ and CZA along with a CDT with CAZ/CZA to recognize ESBL-*Ko*C strains (Table 3). Since avibactam is a potent inhibitor of class A, C and some D β-lactamases (41), we hypothesized that CZA-based confirmatory tests could show more reliable results than those using clavulanate. However, the CDT showed a specificity of 87.5%, while the gradient strip assay resulted in 6 false-positives ESBL producers (specificity, 77.8%). We also noted that, in line with other studies (42), all ESBL-*Ko*C and hOXY-*Ko*C strains resulted in the EUCAST susceptible ranges for CZA (e.g., MIC_90s_ of 0.38 and 0.75 μg/mL, respectively; Table 3) (40).

### Conclusions.

Standard antimicrobial susceptibility profiles for *Ko*C strains can raise some suspicion of ESBL production. However, a clear distinction between ESBL-*Ko*C and hOXY-*Ko*C strains is difficult. With our strain collection, such distinction was achieved only by implementing the FEP/FEP-CL CDT or the DDST-30, whereas all gradient strip- and BMD-based confirmatory tests (regardless of the specific cephalosporin used) did not perform well. It is important to note that the FEP/FEP-CL CDT is not suggested by the CLSI (39), while the EUCAST indicates it only to detect the ESBL production among the group 2 Enterobacteriaceae (species expressing chromosomal AmpC genes) (33). Moreover, the DDST with CAZ, FEP and ATM disks is proposed by the EUCAST, but clear indications about the distance with the AMC disk and the concentration of antibiotics is not yet provided (33).

The CDT with CAZ/CAZ-CL disks may also be implemented as confirmatory tests, but production of OXY variants with potent activity against CAZ (e.g., OXY-2-5) can affect its specificity. In this context, it is worth underlining that the true prevalence of these CAZ-resistant hOXY-*Ko*C strains that may generate phenotypic results identical to those of the CTX-M producers is not known.

In conclusion, the approach to detect contemporary ESBL-*Ko*C strains should not consist on the use of standard gradient strip- or BMD-based confirmatory tests. In contrast, we suggest the simultaneous implementation of FEP/FEP-CL and CAZ/CAZ-CL CDTs or, alternatively, the DDST-30 including at least CAZ, FEP and ATM disks. The use of CAZ (10 μg) and CAZ-CL (10/10 μg) EUCAST-recommended disks seems to perform better than those suggested by the CLSI (30 μg and 30/10 μg, respectively) (33, 39, 40). However, further specific and comparative studies should address this aspect.
