# Supplementary material for: Evaluation of Phenotypic Tests to Detect Extended-Spectrum β-Lactamase (ESBL)-Producing Klebsiella oxytoca Complex Strains
Source: J Clin Microbiol. 2023 Mar 13;61(4):e01706-22. doi: 10.1128/jcm.01706-22 (PMC10117083; doi:10.1128/jcm.01706-22)
Supplement: Supplemental file 1 — Supplemental material. Download jcm.01706-22-s0001.pdf, PDF file, 0.8 MB [file jcm.01706-22-s0001.pdf]

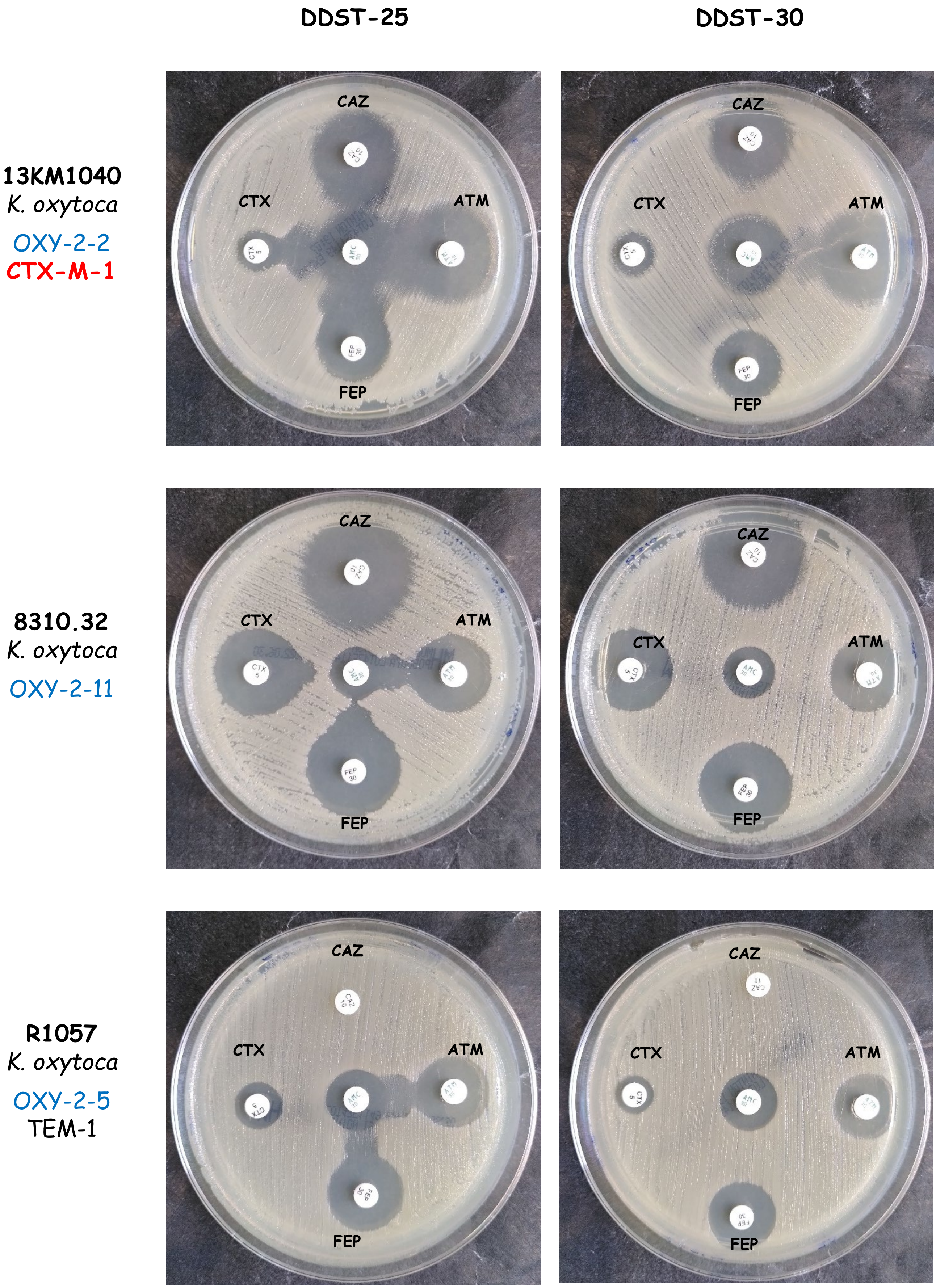

Figure S1. Examples of double-disk synergy tests (DDSTs) performed with the substrates placed with a center-to-center distance with amoxicillin-clavulanate of 25 mm (DDST-25) or 30 mm (DDST-30). Disks: CTX, cefotaxime (5 μg); CAZ, ceftazidime (10 μg); FEP, cefepime (30 μg); ATM, aztreonam (30 μg); AMC, amoxicillin-clavulanate (20/10 μg).

Table S1. Analysis of the promoter region of the *bla*$_{\mathrm{OXY}}$ genes detected in the 44 *K. oxytoca* complex (*Ko*C) strains. Results are grouped in ESBL producers (ESBL-*Ko*C; n=11), hyperproducers of OXY enzymes (hOXY-*Ko*C; n=21), and wildtype (WT-*Ko*C; n=12) strains.

**Strain / species**	**β-lactamase(s)**	**Promoter of *bla*$_{\mathrm{OXY}}$ [a]**
7606.66 / *K. michiganensis*	CTX-M-15, OXY-5-9	GATAGT
7907.29 / *K. michiganensis*	CTX-M-15, OXY-1-2	GATAGT
5401.38 / *K. michiganensis*	CTX-M-15, OXY-1-2, OXA-1, TEM-1	GATAGT
1312240753 / *K. michiganensis*	CTX-M-15, OXY-1-2, OXA-1, TEM-1	GATAGT
8212.48 / *K. oxytoca*	CTX-M-15, OXY-2-12	GATAGT
7407.04 / *K. oxytoca*	CTX-M-15, OXY-2-16, OXA-1	GATAGT
15KM0222 / *K. oxytoca*	CTX-M-1, OXY-2-7	GATAGT
13KM0084 / *K. oxytoca*	CTX-M-1, OXY-2-2, OXA-1	GATAGT
13KM1040 / *K. oxytoca*	CTX-M-1, OXY-2-2	GATAGT
KM57/09 / *K. oxytoca*	CTX-M-1, OXY-2-7	GATAGT
KM24/09 / *K. oxytoca*	CTX-M-1, OXY-2-7	GATAGT
8208.45 / *K. michiganensis*	OXY-1-21	TATAGT
8011.16 / *K. michiganensis*	OXY-1-2	GATAAT
7806.19 / *K. michiganensis*	OXY-1-1	TATAGT
7202.3 / *K. michiganensis*	OXY-1-2	GATAAT
8311.01 / *K. oxytoca*	OXY-2-1	GATAAT
8309.06 / *K. oxytoca*	OXY-2-32	GATAAT
8310.32 / *K. oxytoca*	OXY-2-11	GATAAT
8310.33 / *K. oxytoca*	OXY-2-11	GATAAT
8306.21 / *K. oxytoca*	OXY-2-32	GATAAT
8108.57 / *K. oxytoca*	OXY-2-33	GATAAT
8111.31 / *K. oxytoca*	OXY-2-12	TATAGT
8005.38-1 / *K. oxytoca*	OXY-2-18	GATAAT
8005.38-2 / *K. oxytoca*	OXY-2-18	GATAAT
7510.48 / *K. oxytoca*	OXY-2-10	GATAAT
7610.07 / *K. oxytoca*	OXY-2-1	GATAAT
7707.06 / *K. oxytoca*	OXY-2-34	GATAAT
7802.78 / *K. oxytoca*	OXY-2-4	GATAAT
7907.16 / *K. oxytoca*	OXY-2-6	GATAAT
R1056 / *K. oxytoca*	OXY-2-14, TEM-1	TATACT
R1057 / *K. oxytoca*	OXY-2-5, TEM-1	TATACT
08KM1888 / *K. oxytoca*	OXY-2-16	GATAAT
8310.44 / *K. michiganensis*	OXY-1-20	GATAAT [b]
7507.77 / *K. michiganensis*	OXY-1-1	GATAAT [b]
ZH142-C / *K. michiganensis*	OXY-5-1	GATAGT
17KM0578 / *K. michiganensis*	OXY-1-22, TEM-1	GATAGT
15090013 / *K. michiganensis*	OXY-1-1	GATAGT
15A0136 / *K. michiganensis*	OXY-1-8	GATAGT
20M0142 / *K. grimontii*	OXY-6-4	GATAGT
08KM1900 / *K. grimontii*	OXY-6-4	GATAGT
15Km1352 / *K. pasteurii*	OXY-4-1	GATAGT
17KM1096 / *K. oxytoca*	OXY-2-18	GATAGT
14/F0005 / *K. oxytoca*	OXY-2-2	GATAGT
09KM0284 / *K. oxytoca*	OXY-2-4, TEM-1	GATAGT

[a] The sequences correspond to the -10 (pribnow box) promoter sequence upstream (32-33bp) of the *bla*$_{\mathrm{OXY}}$ gene coding sequence start. Shown in green are the wildtype (WT) promoter; blue, G->T transversion (underlined) in 1st base; orange G->T and G->C transversions (underlined) in first and 5th base, respectively; purple, G->A transversion (underlined) in 5th base.
Note: no mutations in the -35 region or 17bp spacer length were detected.
[b] Besides the -10 promoter sequence, no other nucleotide differences were found between the -35 and -10 promoter regions, including downstream elements such as the transcription start site, ribosome binding site, and Met (start).
